# Evaluation of Cross-Protection of African Swine Fever Vaccine ASFV-G-ΔI177L Between ASFV Biotypes

**DOI:** 10.3390/vaccines13080858

**Published:** 2025-08-13

**Authors:** Manuel V. Borca, Elizabeth Ramirez-Medina, Christine Mutisya, Rose Ojuok, Josiah Odaba, Mark Dihbol, Anna Lacasta, Douglas P. Gladue

**Affiliations:** 1 Plum Island Animal Disease Center, Agricultural Research Service, USDA, Greenport, NY 11944, USA; elizabeth.ramirez@usda.gov (E.R.-M.); mark.dihbol@usda.gov (M.D.); 2 National Bio and Agro-Defense Facility, Agricultural Research Service, USDA, Manhattan, KS 66506, USA; 3 Health Program, International Livestock Research Institute, Nairobi P.O. Box 30709, Kenya; christine.mutisya@cgiar.org (C.M.); rose.ojuok@cgiar.org (R.O.); josiah.odaba@cgiar.org (J.O.); 4 Oak Ridge Institute for Science and Education (ORISE), Oak Ridge, TN 37830, USA

**Keywords:** African swine fever, ASFV, vaccine, cross-protection, I177L, biotypes, live-attenuated vaccines

## Abstract

**Background/Objectives:** Vaccine development for the prevention of ASF has been very challenging due to the extensive genetic and largely unknown antigenic diversity. Inactivated vaccines, using different inactivation methods and a variety of adjuvants, have been consistently inefficacious. Historically, animals recovering from an infection with an attenuated virus became protected from the development of a clinical disease caused by an antigenically related strain. Therefore, immunization of susceptible animals with attenuathe ted virus strains has become a common method of vaccination with the first two commercially available vaccines based on recombinant live-attenuated viruses (LAVs). An important limitation is that the efficacy of the LAV is restricted to those strains that are antigenically related and, in most cases, only provide protection against homologous strains. Due to the unknown antigenic heterogeneity among all ASFV field isolates, the development of broad-spectrum vaccines is a challenge. Besides the anecdotal data, there is not a large amount of information describing patterns of cross-protection between different ASFV strains. **Methods:** We evaluated the cross-protection induced by the ASFV live-attenuated vaccine ASFV-G-ΔI177L against different biotypes of ASFV and compared their genomic sequences to determine potential genetic mutations that could cause the lack of cross-protection. **Results:** Results presented here demonstrate different patterns of protection when ASFV-G-ΔI177L vaccinated pigs were challenged with six different ASFV field isolates belonging to different biotypes. **Conclusions:** The presence of cross-protection cannot be predicted solely by the classical methodology for genotyping-based B646L ORF only. Biotyping, considering the entire virus proteome, appears to be a more promising prediction tool, although additional gathering of experimental data will be necessary to fully validate it; until then, the presence of cross-protection needs to be confirmed in efficacy trials challenging vaccinated animals.

## 1. Introduction

African swine fever (ASF) is a transboundary disease of domestic and wild swine [1]. The clinical presentation of the disease ranges from subclinical to highly lethal depending on the virus strain involved and the characteristics of the host [1]. Historically restricted to South Saharan Africa, the disease is currently present across Eurasia, from Central Europe to East and Southeast Asia [1]. The etiological agent, ASF virus (ASFV), is a large, structurally complex virus harboring a 150–170 Kb double-strand DNA genome which encodes more than 150 proteins [2], with most of these proteins having only a predicted function or protein structure [2,3,4]. In addition to the individual protein analysis, very little has been demonstrated regarding the protein–protein interactions that occur between viral proteins or viral–host interactions. Although the global proteome of ASFV has been evaluated [3,4], it still provides little information as to individual protein complexes.

Vaccine development for the prevention of ASF has been very challenging. Inactivated vaccines, using different types of inactivation methodologies as well as a variety of adjuvants, have been consistently inefficacious [5]. Historically, animals recovering from an infection with an attenuated virus strain became protected from the development of a clinical disease caused by an antigenically related strain [6]. Therefore, immunization of susceptible animals with attenuated virus strains has become a common method of vaccination, at least at the experimental level [6]. Different alternatives have been explored for the development of ASFV-attenuated strains. Naturally attenuated field strains have been experimentally used as a tool to understand the mechanisms of protection induced by live-attenuated vaccines [7,8,9,10,11,12]. Most of these strains present the problem of harboring residual virulence inducing some level of mild, acute, or chronic clinical forms of the disease [11,12,13,14,15]. Several attenuated strains have been developed by adapting virulent field strains to grow in cell cultures [16]. The adaptative process usually produces changes in the virus genome including deletions of large areas of the genome [16]. In addition, most of the attenuated strains produced by this approach also retain a significant level of residual virulence. An alternative approach in the development of attenuated strains is based on the identification of virus genes involved in the process of disease production [6]. The deletion of those genes, by genomic manipulations, produces recombinant virus strains with an attenuated phenotype. These strains are genetically defined, stable, and, when the genetic modifications are correct, lack residual virulence [6]. Several laboratories worldwide have developed different versions of these recombinant viruses based on different genotypes/biotypes, harboring deletions of a variety of genes involved in virulence. In most cases, the resulting recombinant vaccines have proven to be safe and efficacious when evaluated experimentally [6]. In fact, the first two commercial vaccines approved to be used in field conditions are based on recombinant viruses obtained by this approach [17,18,19].

In all these cases, regardless of the methodology used for their development, an important limitation is that the efficacy of the live-attenuated vaccine (LAV) is restricted to those virus strains that are antigenically related. In most cases, these recombinant strains provide protection against their parental virulent strain only [6]. This is a critical bottleneck due to the large genetic and antigenic heterogeneity among all ASFV field isolates, making the development of broad-spectrum vaccines which can be widely used in different geographical areas with different isolates in circulation challenging. Different criteria have been used to classify ASFV isolates in the past: (1) genetically, based on the heterogeneity of single genes, or (2) serologically, based on the cross-reactivity of the antibody response to specific genes, like the EP402R, which mediates the phenomenon of hemadsorption [20]. However, these classifications, grouping different isolates in clusters following specific criteria, are not effective in predicting the presence of cross-protection among field virus strains (reviewed in [21]). Therefore, the only effective way to establish the presence of protective immunity of a particular ASF vaccine among different field isolates is restricted to the actual testing of cross-protection in animal vaccine trials.

Besides anecdotal data, there is not a large amount of information describing patterns of cross-protection between different ASFV strains. In fact, there is a handful of reports experimentally demonstrating specific cross-protection induced by a specific attenuated strain of virus against a virulent non-homologous strain. An attenuated recombinant virus based on the strain Badajoz 1971 (BA71) harboring a deletion of the EP402R gene (BA71ΔCD2) has been described to induce protection against the lethal challenge with the field isolates E75 and Georgia2007 [22]. The BA71 virus belongs to the genotype I, as well as E75, while Georgia2007 is classified within genotype II. Further studies from the same group also demonstrated partial cross-protection against the field isolate RSA/11/2017, which belongs to the genotype XIX but not to Kenya06.Bus, belonging to the genotype IX [23]. Conversely, vaccination with the naturally attenuated field isolate OURT3/88, belonging to the genotype I, failed to induce protection against the challenge with the virulent field isolate MOZ 1/98 (genotype VIII) [24]. Similarly, another naturally attenuated field isolate of genotype II, Lv17/WB/Rie1, although producing partial protection against the virulent isolate Arm07 (also within genotype II), failed to protect animals when challenged with the genotype IX strain Ken06.Bus [25]. A more recent example is constituted by the appearance of a novel virulent field isolate apparently created by the recombination of two field strains belonging to genotypes I and II (rASFV I/II). Recombinant vaccines developed using the genotype II Georgia2010 isolate failed or just produced partial protection in animals challenged with rASFV I/II [26].

Besides the fragmented information obtained in these reports, the diverse methodologies used in each case, including vaccine dosage, immunization schedule, route of administration, characteristics of the animals employed in the experiments, dosage, and route of administration of the challenge virus make it difficult to draw strong conclusions.

In this report, we present data on a series of systematically conducted challenge experiments, performed under standardized experimental conditions, where we evaluated the efficacy of a well-characterized recombinant vaccine strain, ASFV-G-ΔI177L, to protect domestic pigs against six genetically different virulent ASFV field strains. We analyzed the variations in protection outcomes in relation to the genetic characteristics of the challenge virus, drawing comparisons with both the classical and recently proposed concepts of genotyping [27], as well as the novel notion of biotyping [28].

## 2. Materials and Methods

### 2.1. Cell Culture and Viruses

Primary swine macrophage cultures were prepared from defibrinated swine blood, as previously described [29]. Development of the live-attenuated vaccine candidate strain ASFV-G-ΔI177L has been previously described [17]. Georgia10, Ghana2021, Uganda1965, Malawi Lil20/1, and Pretoriuskop/96/4 (Pret4) ASFV strains were bulked-up on blood macrophages. The Kenya1033 ASFV strain was bulked-up on PAMs isolated from the lungs of healthy and ASFV-free animals (also tested for the presence of bacterial and fungal infections by cell culture).

The Georgia10 ASFV strain was kindly donated by Dr. Nino Vepkhvadze, from the Laboratory of the Ministry of Agriculture (LMA) in Tbilisi, Republic of Georgia, virus strains Uganda1965, Malawi Lil20/1, and Pretoriuskop/96/4 belong to the Plum Island Animal Disease Center collection. The Kenya1033 ASFV strain belongs to the ILRI strain collection [30]. Ghana2021 was kindly donated by Dr. Theophilus O’Doom from the Ministry of Agriculture, Accra, Ghana [31].

Virus titrations were performed in PAMs as described earlier [29], the presence of the virus was assessed by hemadsorption (HAD), and the virus titers were calculated by the Reed and Muench Method [32].

### 2.2. Assessment of Virus Replication

Comparative growth curves between the different challenge viruses were performed in PAMs from an ASFV-free donor pig. PAMs at a concentration of 5 × 10^5^ cells/well were precultured 24 h in advance in 96-well plates and infected at an MOI of 0.1 (based on HAD_50_ previously determined in PAM cultures). After 2 h of adsorption at 37 °C under 5% CO_2_, the inoculum was removed, and the cells were rinsed two times with PBS. The monolayers were then rinsed with macrophage media (composed by RPMI supplemented with 10% heat-inactivated FBS (Gibco), 2 mM L-glutamine, 100 μg/mL streptomycin, and 100 UI/mL penicillin (all from Merck)) and incubated for 2, 6, 24, 48, 72, and 96 h at 37 °C under 5% CO_2_. At appropriate times post-infection, the cells were frozen at −70 °C, and the thawed lysates were used to determine titers by B646L qPCR [13]. All samples were run simultaneously to avoid inter-assay variability. The results are presented as genomic equivalent copies per milliliter of sample (GEC/mL).

### 2.3. Animal Experiments

Duroc x Landrace x Large White domestic pigs (*Sus scrofa domesticus*) of around twelve to sixteen weeks of age which were negative for ASFV antibodies and virus in their blood were sourced from Kenyan pig farms from ASFV-free areas. Animals were vaccinated against Foot-and-Mouth Disease (FMD) three weeks before the start of the experiment, following the ILRI farm policy. Animals were clinically assessed by the ILRI Institutional Veterinarian and acclimatized to the ABSL-2 facilities for at least 5 days before starting the experiment. Animal experiments and routine maintenance were in accordance with procedures approved by ILRI’s Institute Animal Care and Use Committee with references: IACUC2022-46 (approved 27 January 2023), IACUC2023-12 (approved 5 July 2023), and IACUC2024-04 (approved 11 April 2024).

Each experiment consisted of twelve (12) animals divided into two (2) groups of six (6) animals. Groups were balanced by sex and weight. The experimental groups were blinded by the ILRI Data and Research Methods Unit (DRMU). In each experiment, one group was vaccinated intramuscularly with a single dose of 10^4^ HAD_50_ of ASFV-G-ΔI177L, and another group was mock-vaccinated with PBS. Twenty-eight days after vaccination five animals per group were intramuscularly challenged with a lethal dose 100 (LD100) of highly virulent African swine fever virus: Experiment 1 (Georgia2010 at 10^2^ HAD_50_), Experiment 2 (Ghana2021 at 10^2^ HAD_50_), Experiment 3 (Malawi Lil20/1 at 10^2^ HAD_50_), Experiment 4 (Pretoriuskop/96/4 at 10^4^ HAD_50_), Experiment 5 (Kenya1033 at 10^2^ HAD_50_), and Experiment 6 (Uganda1965 at 10^2^ HAD_50_). The selection of the virus was based on the representation of field isolates for each biotype and with epidemiological relevance. One vaccinated and one mock-vaccinated pig were removed from the experiment before challenge at day 26/27 for post-mortem evaluation of lesions related to vaccination with ASFV-G-ΔI177L, leaving the groups with 5 animals to be challenged. Clinical signs including temperature, recumbency, inappetence, skin hemorrhage, joint swelling, breathing difficulties, diarrhea, vomiting, and blood in urine were recorded daily, and clinical scores were calculated after vaccination and after challenge, as previously described [13].

### 2.4. Real-Time Quantitative PCR for the Detection of ASFV Genome

Presence of virus genome in EDTA-blood and tissues was evaluated by qPCR as previously described [13,33], with minor modifications. Briefly, genomic DNA was isolated using a DNeasy blood and tissue extraction kit (Qiagen, Venlo, The Netherlands), from 100 μL of EDTA-blood 1 in 2 in PBS at the time of sampling. Each qPCR reaction was conducted in duplicates in a 25 μL reaction containing 7.9 μL of nuclease-free water, 12.5 μL of TaqMan Fast Advanced Master Mix (Applied Biosystems, Foster City, CA, USA), 0.8 μL/each of forward and reverse primer at 10 μM/each, 1 μL of probe at 5 μM, and 2 μL of sample. The qPCR conditions, primers, and probe sequences are the same as previously described [13]. qPCRs were performed using QuantiStudio™ 5 system (Applied Biosystems). The Ct value of the unknown samples was measured and compared to the standard curve with a known number of copies, built with plasmid DNA encoding a section of B646L, as previously described [33]. Results are expressed as genome-equivalent copies per milliliter (GEC/mL) of EDTA-blood sample.

### 2.5. Detection of Anti-ASFV Antibodies

Anti-ASFV antibody titers were detected by means of in-house indirect ELISA. ASFV antigen was prepared by infection of WSL cells with Kenya1033 WSL-adapted ASFV at an MOI of 10 for a maximum of 48 h at 37 °C and 5% CO_2_. After incubation, infected cells were treated with lytic buffer containing 25 mM Tris (Merk, Darmstadt, Germany), 150 mM NaCl (Merk), 1% Triton X-100 (Merk), and proteinase inhibitor tablets (Pierce, Rockford, IL, USA) for 10 min at room temperature while shaking. Lysed material was vortexed vigorously for 30 s and spun down at 16,000 g for 10 min at 4 °C. Finally, supernatant was collected, and protein content was quantified using the BCA Protein Assay kit (Pierce) and used as a coating antigen.

Maxisorp 96-well plates (Nunc) were coated with 1 μg/mL of ASFV antigen diluted in PBS (Merk) and incubated at 4 °C overnight. After blocking the plates for 1 h at 37 °C with blocking buffer (PBS complemented with 1% bovine serum albumin (BSA, Merk, and 0.1% Tween20 (Merk)), sera serially diluted 2-fold in blocking buffer from 1 in 100 to 1 in 102,400 were added to the plate and incubated for 1 h at 37 °C. The presence of ASFV-specific antibodies was detected using polyclonal rabbit anti-pig IgG peroxidase conjugated (Merk) diluted at 1 in 20,000 in blocking buffer and incubated for 1 h at 37 °C. The reaction was developed with 50 μL of TMB plus 2 (Kem-En-Tec Diagnostics, Taastrup, Denmark) in the dark for 10 min at room temperature and stopped using 50 μL of 0.5 M H_2_SO_4_ (Honneywell-Fluka, Morris Plains, NJ, USA). Plates were washed four times in between steps using PBS containing 0.05% Tween20, and assays were carried out in duplicates. The optical density at 450 nm was read using Synergy HT ELISA reader (BioTek Instruments, Winooski, VT, USA). The area under the receiver operating characteristic (ROC) curve was calculated by comparing the results from the present ELISA with the commercial competitive ELISA INgezim PPA COMPAC (Gold Standard Diagnostics, Davis, CA, USA). The threshold where the sensitivity and specificity were balanced was selected for every dilution of sera. The area under the curve (AUC) was higher than 80% for all the dilutions tested. The analysis of ROC and AUC were performed using the R (version 2024.12.0+467) “pROC” package.

### 2.6. Analysis of Cellular Response to the Vaccine ASFV-G-ΔI177L by IFNg-ELIspot

IFNγ-ELIspot for the analysis of PBMC response to the vaccine ASFV-G-ΔI177L was performed as previously described [33]. Briefly, ELIspot plates (Millipore, MAHAS4510) were coated at 4 °C overnight with mouse anti-pig IFNγ clone P2G10 (BD Pharmingen, Franklin Lakes, NJ, USA) diluted at 0.5 μg/mL. Before adding the cell stimuli, the plates were blocked for 2 h at room temperature with 4% skimmed milk (Marvel, Premier Foods group, Thame, UK) diluted in PBS. Stimuli used for this experiment were as follows: Concanavalin A at 10 μg/mL (Merk) and ASFV-G-ΔI177L at MOI of 1. Media was used as a negative control for stimulation and to set the background response of individual animals. PBMCs (5 × 10^5^ cells/well) were seeded in the respective wells, and plates were incubated for 20 h at 37 °C and 5% CO_2_. Assays were carried out in duplicates. Biotin mouse anti-pig IFNγ clone P2C11 (BD Pharmingen) diluted at 0.25 μg/mL in PBS was added and incubated for 2 h at room temperature and followed by a 1 h incubation with streptavidin alkaline phosphatase (Invitrogen) at 2 μg/mL in PBS. The reaction was developed using SIGMAFAST BCIP/NBT substrate (Merk) and incubated for 20 min in the dark. The spot-forming units were counted using the AID classic ELISPOT reader (AID AutoImmun Diagnostika GmbH, Straßberg, Germany). Results are reported as spot-forming units (SFU) per million of PBMCs.

### 2.7. Statistical Analysis

Response to vaccination was calculated as the duration of fever or viremia after vaccination and before challenge. All models are included in the “stats” package version 3.6.2.

A Kaplan–Meier curve was used to evaluate the probability of surviving over time of the different groups. The “survival” (version 3.6.4) and “survminer” (version 0.5.0) packages were used for this analysis.

The temperature, clinical scores, and viremia progression over time was evaluated using a linear mixed effect model where the group, time, experiment, sex, and weight at the initial examination were included as fixed effects and animals as random effects (“lme4” version 1.1.35.5 and “emmeans” version 1.10.4 packages were used for this analysis). Temperature, clinical scores, and viremia were assessed for normality and equal variances of the sample using a residual model check. All tests were performed using R-studio version2024.12.0+467.

## 3. Results

### 3.1. Comparative Growth Kinetics of the Challenge Viruses

**Figure 1:**
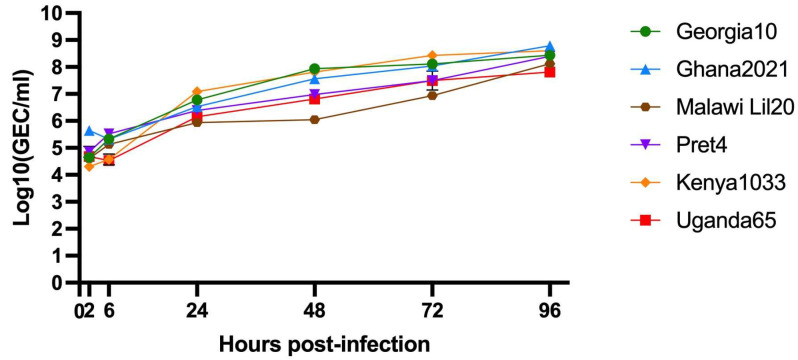
In vitro growth kinetics in primary swine macrophage cell cultures for ASFV Georgia2010, Ghana2021, Malawi Lil20, Pret4, Kenya1033, and Uganda65 at MOI of 0.1. Data represents means and standard deviations of six replicates.

To assess the replicative ability of each of the ASFV strains used in the challenge experiments, the replication kinetics of these viruses were comparatively evaluated in swine pulmonary alveolar cell cultures. A multistep growth curve was implemented, infecting swine macrophage cultures with an MOI of 0.1 with each of the virus strains under study and virus yields assessed at 2-, 6-, 24-, 48-, 72-, and 96-h pi. Results showed that the growth kinetics of all viruses assessed practically overlap without showing statistically significant differences among them in any of the evaluated time points, when tested in primary cultures of swine macrophages, the natural cell host during the infection in domestic pigs (Figure 1).

### 3.2. Protection Induced by ASFV-G-ΔI177L Against the Homologous Challenge

The experimental conditions to assess the presence of homologous and heterologous protection were first set by establishing the efficacy of ASFV-G-ΔI177L in protecting domestic pigs against the challenge with the homologous parental strain ASFV Georgia2010 (ASFV-G). It has already been reported that ASFV-G-ΔI177L efficaciously protects domestic pigs against the challenge with the homologous strain ASFV-G or its derivatives in a dose range of 10^2^ to 10^6^ HAD_50_ [19,21]. In this report, a vaccine dose of ASFV-G-ΔI177L 10^4^ HAD_50_ was selected to minimize potential variability in the vaccination procedures and to closely match the actual dosage of the ASFV-G-ΔI177L vaccine in its commercial presentation form [18].

**Figure 2:**
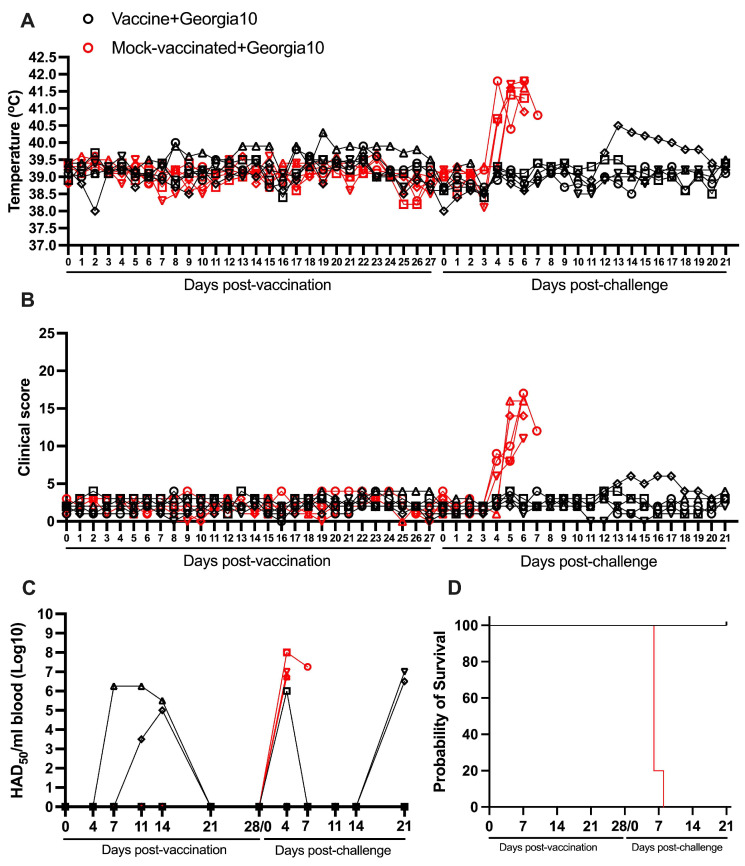
Parameters obtained in pigs IM vaccinated with 10^4^ HAD_50_ of ASFV-G-∆I177L and challenged 28 days later IM with 10^2^ HAD_50_ of ASFV strain Georgia10. (**A**) Evolution of body temperature, (**B**) clinical scores, (**C**) viremia titers (sensitivity of virus detection: >log10^1.8^ HAD_50_/mL), and (**D**) evolution of mortality. In panels (**B**–**D**) each symbol represents individual values for each animal in either group.

A group of six animals was IM inoculated while another group with similar characteristics was mock-vaccinated. The clinical observation of both groups of animals conducted on daily bases during a period of 28 days post-vaccination (pv) demonstrated the absence of any clinical sign that may be associated with ASF. Body temperature readings remained below or at the top physiological value of 40 °C in all animals in both groups with the exception of one animal in the vaccinated group, which presented a slight and transient increase on day 19 pv. Kinetics of body temperature and clinical scores in vaccinated and mock-vaccinated animals are very similar during the 28-day-pv observational period (Figure 2A,B).

The absence of any clinical abnormality in ASFV-G-ΔI177L inoculated animals coincides with results previously reported [17]. Viremia detected by hemadsorption demonstrated heterologous patterns in the vaccinated animals with titers ranging from undetectable to 10^6.25^ HAD_50_/mL blood. The vaccine virus was only detectable in two of the six vaccinated pigs (Table 1). All vaccinated animals showed undetectable levels of virus by day 21 pv (Figure 2C).

Both groups of animals were IM challenged with 10^2^ HAD_50_ of the homologous virulent parental virus ASFV-G, belonging to Biotype 2 [17]. Again, the presence of clinical signs and body temperature were monitored daily during a 21-day period. As expected, all mock-vaccinated animals presented a sudden increase in body temperature by day 4–5 post challenge (pc) with a rapid progressions in the severity of clinical disease that resulted in the euthanasia of all animals between day 5 and 8 pc (Figure 2A,B,D and Table 2).

Conversely, all animals vaccinated with ASFV-G-ΔI177L remained clinically healthy until the end of the 21-day observational period. Only one animal presented a mild transitory increase in body temperature (never higher than 40.5 °C) on days 13 to 16 pc. This animal did show a slight and transitory episode of vomit on day 14 pc (Figure 2A,B).

Viremias in the mock-vaccinated group appeared from day 4 pc in all animals, with values ranging from 10^6.75^ to 10^8^ HAD_50_/mL blood. In the vaccinated animals, viremia values after challenge also presented some degree of heterogenicity. One animal presented transient viremia on day 4 pc (10^6^ HAD_50_/mL blood) and two other animals did not present any detectable virus until day 21 pc with titers of 10^6.5^ to 10^7^ HAD_50_/mL blood (Figure 2C).

### 3.3. Heterologous Protection Induced by ASFV-G-ΔI177L Against an ASFV Strain from the Biotype 1 (Ghana2021)

**Figure 3:**
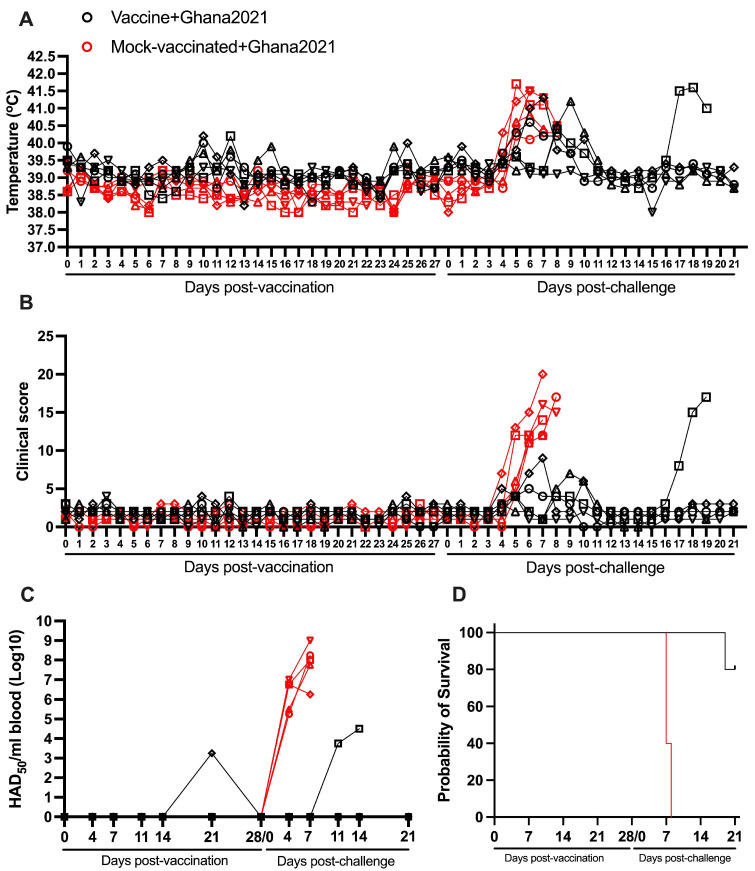
Parameters obtained in pigs IM vaccinated with 10^4^ HAD_50_ of ASFV-G-∆I177L and challenged 28 days later IM with 10^2^ HAD_50_ of ASFV strain Ghana2021. (**A**) Evolution of body temperature, (**B**) clinical scores, (**C**) viremia titers (sensitivity of virus detection: >log10^1.8^ HAD_50_/mL), and (**D**) evolution of mortality. In panels (**B**–**D**) each symbol represents individual values for each animal in either group.

As in the previous experiment, animals receiving an IM dose of 10^4^ HAD_50_ of ASFV-G-ΔI177L showed the absence of any clinical sign associated with ASF. Evaluation of body temperature demonstrated that all animals remained below or at the top physiological value of 40 °C with the exception of two vaccinated animals which presented a slight and transient increase (never higher than 40.5 °C), one of them on days 10–11 pv and the other on day 12 (Figure 3A and Table 1).

None of these animals presented any clinical signs associated with ASF (Figure 3B). The kinetics of body temperature and, particularly, that of the clinical scores in mock-vaccinated animals are similar to those of the vaccinated animals during the 28-day pv observational period (Figure 3A,B). As in the first experiment, viremias showed heterogenous patterns. Only one of the animals presented a low detectable titer (10^3.21^ HAD_50_/mL) on day 21 pv, while all blood samples from the other four animals retained undetectable levels of infectious virus during the 28 days pv. All vaccinated animals presented undetectable levels by the challenge day (Figure 3C).

After the IM challenge with 10^2^ HAD_50_ of virulent virus ASFV strain Ghana2021, all mock-vaccinated animals presented a rapid increase in body temperature since day 5 pc followed by a quick and severe form of clinical disease, with all animals being euthanized between day 7 and 8 pc (Figure 3A,B,D and Table 2). One of the five animals in the group vaccinated with ASFV-G-ΔI177L remained clinically normal until the end of the 21-day pc observational period. The other four animals presented a transient elevation in body temperature (ranging between 40.5 and 41 °C) around days 6 to 9 pc, accompanied by mild depression and decreased appetite. Three of these animals returned to normal body temperature values and clinical scores by day 10 pc and remained clinically normal until the end of the observational period (21 days pc). The remaining animal presented an additional sudden increase in body temperature accompanied by clinical disease, by day 17 pc, that worsened in the following days, being euthanized by day 19 pc (Figure 3A,B,D). As expected, viremias in the mock-vaccinated group were detected at day 4 pc with values ranging from 10^5.25^ to 10^7^ HAD_50_/mL. In the vaccinated animals, viremia values after challenge remained undetectable in all animals surviving the challenge. Viremia was only detected in the unprotected animal with titers ranging between 10^3.75^ and 10^4.5^ HAD_50_/mL (Figure 3C). Therefore, 80% of the vaccinated animals survived the challenge with a virulent virus belonging to the Biotype 1 (ASFV strain Ghana2021) only presenting a transient and mild clinical disease and reaching the end of the observational period in good clinical conditions.

### 3.4. Protection Induced by ASFV-G-ΔI177L Against a ASFV Strain from the Biotype 6 (Malawi Lil20)

**Figure 4:**
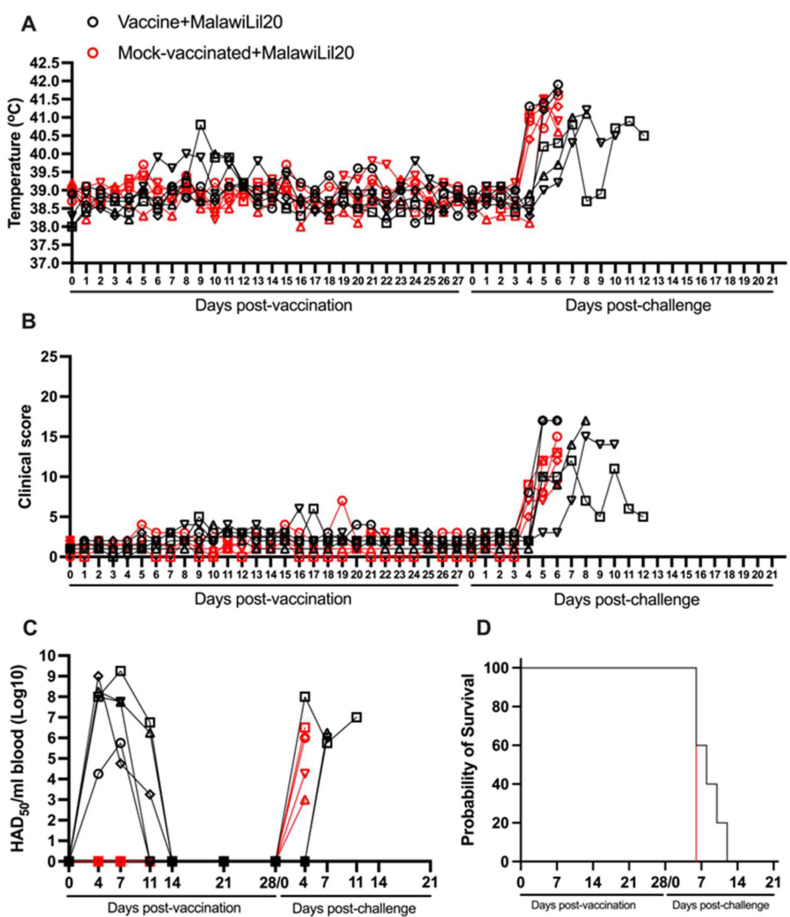
Parameters obtained in pigs IM vaccinated with 10^4^ HAD_50_ of ASFV-G-∆I177L and challenged 28 days later IM with 10^2^ HAD_50_ of ASFV strain Malawi Lil20. (**A**) Evolution of body temperature, (**B**) clinical scores, (**C**) viremia titers (sensitivity of virus detection: >log10^1.8^ HAD_50_/mL), and (**D**) evolution of mortality. In panels (**B**–**D**) each symbol represents individual values for each animal in either group.

Animals vaccinated with one IM dose of 10^4^ HAD_50_ of ASFV-G-ΔI177L showed no major clinical signs associated with ASF. Only one of the vaccinated animals showed a slight and transient increase (no higher than 40.5 °C) on day 9 pv, unassociated with any ASF clinical sign. Body temperature and clinical scores kinetics in mock-vaccinated animals were similar to those of the vaccinated animals during the 28-day pv observational period (Figure 4A,B).

Viremias in the vaccinated animals showed homogenous patterns. All animals had titers with values ranging from 10^3.25^ to 10^9.25^ HAD_50_/mL in almost all samples taken during the first 14 days after vaccination. All vaccinated animals presented undetectable viremia levels by the challenge day at 28 days pv (Figure 4C).

After the IM challenge with 10^2^ HAD_50_ of virulent virus ASFV strain Malawi Lil20, all mock-vaccinated animals developed clinical disease starting at day 5–6 pc with a rapid worsening of the clinical disease with all animals being euthanized by day 6 pc (Figure 4A,B,D and Table 2). The vaccinated animals presented a more heterogeneous pattern. Two of them showed disease kinetics similar to the mock-vaccinated ones with a quick onset and evolution of clinical disease, being euthanized on day 6 pc. A third animal presented a disease onset on day 5, evolving to a severe form of the disease and euthanasia on day 8 pc. The other two animals presented a relatively late disease onset by day 7 pc with disease kinetics that lead progressively to a severe form of clinical disease at days 10 and 12 pc, respectively (Figure 4A,B,D). All mock-vaccinated animals showed viremia titers by day 4 pc with values ranging from 10^3^ to 10^6.5^ HAD_50_/mL. Also, all vaccinated animals presented viremia levels between 10^5.75^ and 10^8^ HAD_50_/mL by the day they were euthanized (Figure 3C). Therefore, it appears that vaccination with ASFV-G-ΔI177L provoked a protracted presentation of the disease as produced by the virulent Malawi isolate in some of the animals; with statistical differences in the survival curves (*p*-value 0.0495) and in the progression of the viremia (*p*-value 0.0176) compared to the control group.

### 3.5. Protection Induced by ASFV-G-ΔI177L Against a ASFV Strain from the Biotype 3 (Pret4)

**Figure 5:**
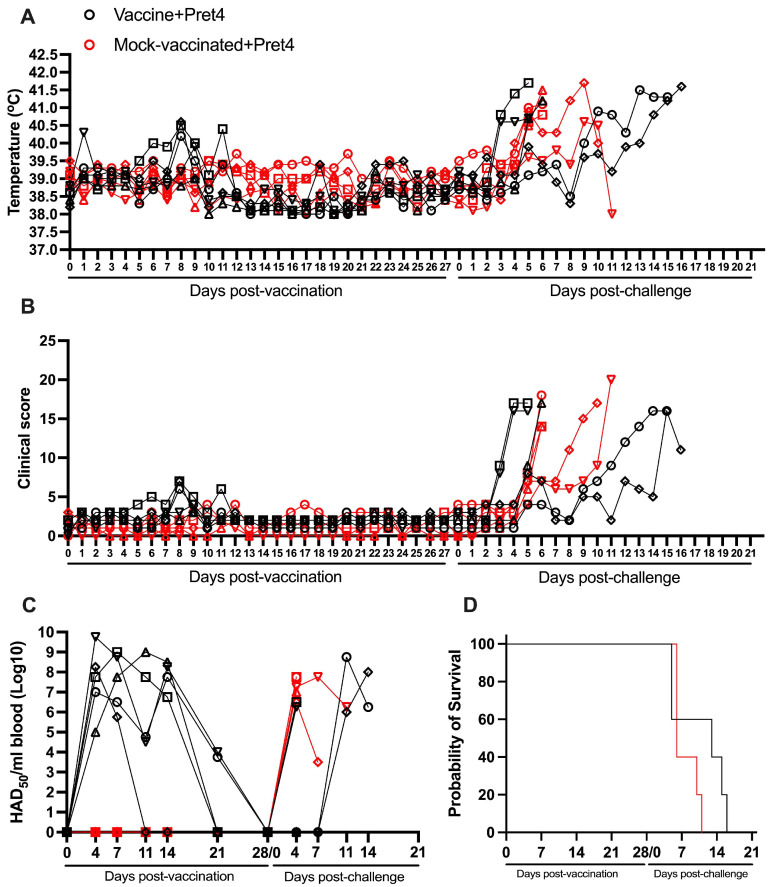
Parameters obtained in pigs IM vaccinated with 10^4^ HAD_50_ of ASFV-G-∆I177L and challenged 28 days later IM with 10^2^ HAD_50_ of ASFV strain Pret4. (**A**) Evolution of body temperature, (**B**) clinical scores, (**C**) viremia titers (sensitivity of virus detection: >log10^1.8^ HAD_50_/mL), and (**D**) evolution of mortality. In panels (**B**–**D**) each symbol represents individual values for each animal in either group.

All vaccinated animals remained clinically normal after the IM inoculation of 10^4^ HAD_50_ of ASFV-G-ΔI177L with the exception of transient and mild increases in body temperature (never higher than 40.5 °C) in three animals at days 1, 8, and 11 pv, respectively. None of these body temperature increases were accompanied by clinical signs associated with ASF. Body temperature values and clinical scores in the mock-vaccinated animals were within the range of the values observed in animals in the vaccinated group (Figure 5A,B). Viremias were detected in almost all animals during all the pv observational periods showing titers ranging between 10^3.75^ and 10^9.75^ HAD_50_/mL. All animals showed undetectable levels by day 28 pv (Figure 5C and Table 1).

After the IM challenge with 10^4^ HAD_50_ of virulent virus ASFV strain Pret4, animals in both groups presented heterogenous behavior. Three of the non-vaccinated animals presented a quick onset of a severe form of the disease, leading to the euthanasia of all animals by day 6 pc. The other two animals developed a delayed form of severe disease starting by day 8 pc leading to the euthanasia of both of them by days 10 and 11 pc, respectively (Figure 5A,B,D and Table 2). Two of the vaccinated animals showed disease kinetics similar to the mock-vaccinated ones, experiencing a quick onset and evolution of clinical disease and being euthanized on days 5 and 6 pc. A third animal presented a protracted disease onset, being euthanized on day 12 pc. The last two animals presented a late disease onset, by days 10 and 14 pc, experiencing severe disease kinetics leading to their euthanasia at days and 16 pc, respectively (Figure 5A,B,D). All mock-vaccinated animals showed viremia 15 titers by day 4 pc with values ranging from 10^6.5^ to 10^7.75^ HAD_50_/mL, while all vaccinated animals presented viremia levels between 10^6^ and 10^8.75^ HAD_50_/mL by the day they were euthanized (Figure 5C). The overall evolution of viremia titers in vaccinated and non-vaccinated animals was significantly different (*p*-value 0.083).

Therefore, as in the case of Malawi, vaccination with ASFV-G-ΔI177L induced a delayed presentation of the lethal form of disease in some of the animals when challenged with the virulent Pret4 isolate.

### 3.6. Protection Induced by ASFV-G-ΔI177L Against an ASFV Strain from the Biotype 5 (Kenya1033)

**Figure 6:**
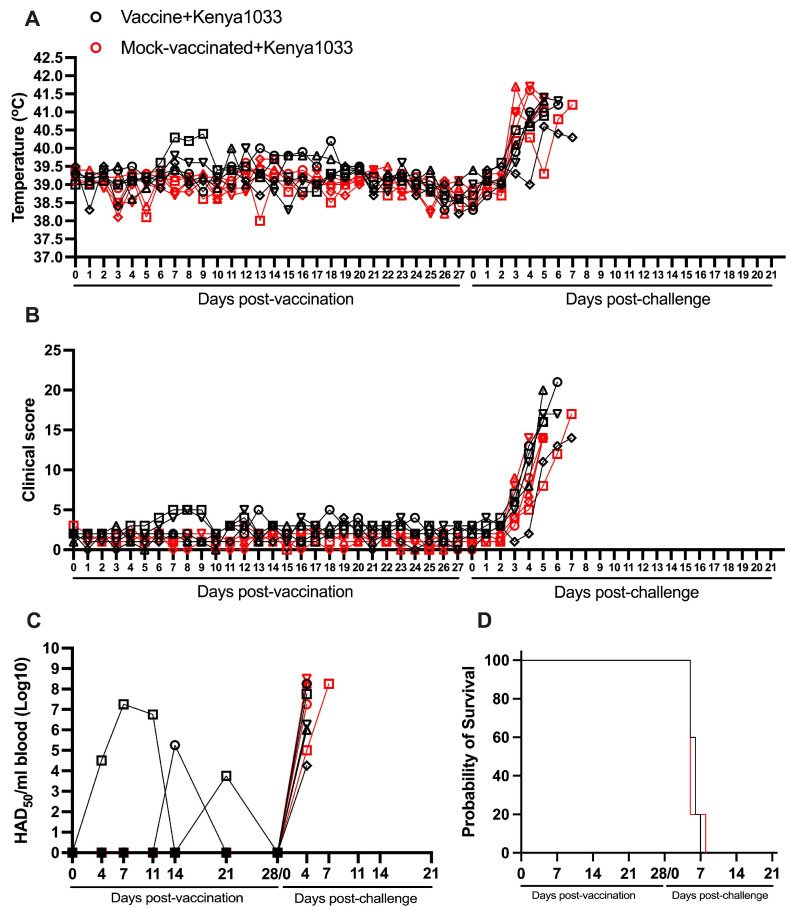
Parameters obtained in pigs IM vaccinated with 10^4^ HAD_50_ of ASFV-G-∆I177L and challenged 28 days later IM with 10^2^ HAD_50_ of ASFV strain Kenya1033. (**A**) Evolution of body temperature, (**B**) clinical scores, (**C**) viremia titers (sensitivity of virus detection: >log10^1.8^ HAD_50_/mL), and (**D**) evolution of mortality. In panels (**B**–**D**) each symbol represents individual values for each animal in either group.

All animals IM inoculated with 10^4^ HAD_50_ of ASFV-G-ΔI177L remained clinically normal during the 28-day observation period, with the exception of transient and mild increases in body temperature (never higher than 40.5 °C) in one animal by days 7 to 9 pv. This transitory increase in body temperature was not accompanied by clinical signs associated with ASF. Body temperature and clinical score values in the mock-vaccinated group do not substantially differ from the vaccinated animals. (Figure 6A,B).

Viremias in two of the vaccinated animals were detected intermittently by day 4–28 pv (with levels between 10^3.75^ and 10^7.25^ HAD_50_/mL), with all animals showing undetectable levels by day 28 pv. The other three remaining animals presented undetectable viremia values during the entire 28-day pv period (Figure 6C and Table 1).

All mock-vaccinated animals developed a severe form of clinical disease after they were IM challenged with 10^2^ HAD_50_ of the virulent virus ASFV strain Kenya1033. All animals presented a marked increase in body temperature by day 3–4 pc followed by a severe clinical form of the disease and all animals being euthanized between days 6 and 8 pc (Figure 6A,B,D and Table 2).

After the challenge, the group of vaccinated animals developed a clinical kinetic disease similar to that of the mock-vaccinated group. Their onset of disease was between 3 and 4 days pc with all animals needing to be euthanized due to the severity of the clinical signs between days 6 and 8 pc. Viremias in both groups of animals showed all high titers (with values between 10^4.25^ and 10^8.5^ HAD_50_/mL), which remained high until they were euthanized (Figure 6C). Therefore, vaccination with ASFV-G-ΔI177L does not induce any protection in animals challenged with the virulent isolate Kenya1033.

### 3.7. Protection Induced by ASFV-G-ΔI177L Against an ASFV Strain from the Biotype 4 (Uganda65)

**Figure 7:**
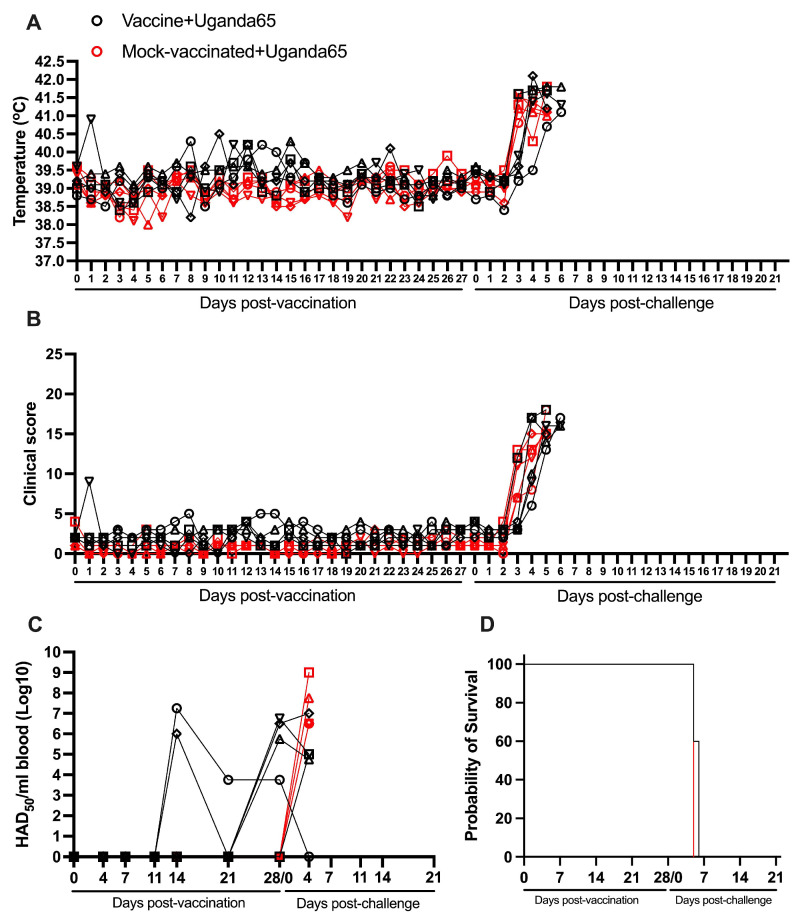
Parameters obtained in pigs IM vaccinated with 10^4^ HAD_50_ of ASFV-G-∆I177L and challenged 28 days later IM with 10^2^ HAD_50_ of ASFV strain Uganda65. (**A**) Evolution of body temperature, (**B**) clinical scores, (**C**) viremia titers (sensitivity of virus detection: >log10^1.8^ HAD_50_/mL), and (**D**) evolution of mortality. In panels (**B**–**D**) each symbol represents individual values for each animal in either group.

All animals vaccinated with 10^4^ HAD_50_ of ASFV-G-ΔI177L remained clinically normal during the first 28 days after inoculation, although transient and mild increases in body temperature (oscillating between 40.0 and 40.5 °C) were observed in some animals between days 8 and 15 pv (Figure 7A,B and Table 2).

These rises in body temperature were accompanied by very mild episodes of depression in two of the animals. Again, body temperature and clinical score values in the mock-vaccinated group do not practically differ from those in the group of vaccinated animals. Viremias in all vaccinated animals were detected since day 11 (with levels between 10^3.75^ and 10^7.25^ HAD_50_/mL) at different times pv, with four of the animals presenting detectable viremias (10^3.75^ to 10^6.75^ HAD_50_/mL) at the time of challenge (Figure 7C and Table 1).

After the IM challenge with 10^2^ HAD_50_ of virulent virus ASFV strain Uganda65, both groups of animals, vaccinated and mock-vaccinated, developed a severe clinical disease practically undistinguishable between treatments. All mock-vaccinated animals showed high body temperature by day 3 pc, with all of them euthanized by day 6 pc (Figure 7A,B,D and Table 2). The vaccinated ones presented a disease onset between days 3 and 5 pc, with two animals euthanized on day 5 pc and the remaining three euthanized by day 6 pc. Viremias in all animals in both groups were detected by day 4 pc (with levels between 10^4.75^ and 10^9^ HAD_50_/mL), remaining at that level until their euthanasia (Figure 7C). Therefore, as in the case of animals challenged with virulent isolate Kenya1033, vaccination with ASFV-G-ΔI177L does not protect animals against the challenged with virulent isolate Uganda65.

### 3.8. Detection of ASFV Specific Immune Response in All Groups Vaccinated with ASFV-G-ΔI177L

**Figure 8:**
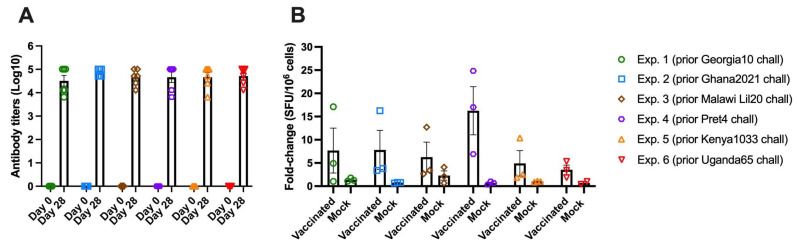
Immune response detected in pigs at 28 days after being vaccinated with 10^4^ HAD_50_ of ASFV-G-∆I177L. Detection of ASFV-specific antibody (**A**) and cellular (**B**) response detected by ELISA and IFNγ–ELISPOT, respectively. Data is presented as average as well as the individual data for each animal in the group.

To ensure all animals vaccinated with ASFV-G-ΔI177L in each of the experiments developed a comparable immune response, the levels of ASFV-specific antibodies and cellular response (IFNγ) were assessed. Titers of virus-specific antibody response were assessed is every vaccinated animal. The IFNγ-producing cells in blood of a representation of three animals in each group, vaccinated and mock-vaccinated, were determined at the time of challenge. All ASFV-G-ΔI177L-vaccinated animals in all the experiments developed strong virus-specific antibody responses, detected by ELISA, with quite homogenous high values over log 10^−4^ dilution of serum (Figure 8A).

Similarly, the measurement of virus-specific IFNγ-secreting cells in all those animals produced comparable results at the time of challenge (Figure 8B). Therefore, the homogeneity in the levels of the immune response induced by the vaccination with ASFV-G-ΔI177L indicates that there was a consistency in the vaccine efficacy across all the experiments described in this report.

## 4. Discussion

The results obtained during the challenge trials were analyzed to establish a potential correlation with the genetic characteristics of the viruses involved in the experiments. Thus, a comparison of the full-length genomic nucleotide sequence of ASFV-G-ΔI177L was performed with that of all viruses used in the challenge experiments (Supplementary Table S1).

Traditional genotyping using the nucleotide sequence of a partial area of the gene encoding for p72 was never intended to predict cross-protection after vaccination, but at the time, it was a method for easy gene tracking when next-generation sequencing was not yet possible [34]. Recently, genotyping was re-evaluated by using the full-length amino acid sequence of p72 to bring the classification between isolates to a higher level of accuracy [27]. The first interesting observation is that there is no cross-protection among viruses that belong to the same genotype based on the amino acid composition of p72, suggesting that the role of p72 in cross-protection prediction has very minimal value. Both ASFV-Georgia2010 and Pret4 viruses have identical p72 amino acid sequences; therefore, both are classified as genotype II, but ASFV-G-ΔI177L does not induce any protection against the challenge with Pret4. Therefore, genotyping based on the p72 sequence fails in predicting the presence of cross-protection between different ASFV isolates. In addition, predicting cross-protection between isolates by analyzing potential differences in nucleotide sequence when the amino acid sequence is 100% conserved is not scientifically sound. Recently, a novel classification of ASF field isolates was proposed based on comparisons performed considering the entire encoded proteome [28]. In this regard, it should be mentioned here that ASFV-Georgia2010 and Pret4 belong to different Biotypes, 2 and 3, respectively, when the amino acids from all ASFV genes are taken into account using Biotyping classification [28].

The observed cross-protection induced by ASFV-G-ΔI177L (Biotype 2) and ASFV-Ghana2021 (Biotype 1) showing that vaccination with a Biotype 2 ASFV strain induced 80% protection against the challenge with Biotype 1 is an interesting result, particularly because Biotype 2-based vaccines do not protect animals against the Biotype 1-2 hybrid field isolate in China, ASFV-Henin/2022 [26]. The computational analysis of the amino acid differences between ASFV-Georgia2010 (the parental virus for ASFV-G-ΔI177L) and ASFV-Ghana2021 shows that there is at least one amino acid difference in 148 individual viral proteins. When the same analysis is conducted between the Biotype 1-2 hybrid virus ASFV-Henin/2022 and the ASFV-Georgia isolate, only 74 proteins showed amino acid differences between these two isolates, as expected, since this virus is a hybrid containing nucleotide sequences from an ASFV-Georgia derivative virus circulating in China. Of the 74 proteins that were different, 73 of these proteins also had amino acid differences between ASFV-Georgia and the ASFV-Ghana isolate. However, it should be mentioned that there are some notable differences. ASFV protein C122R has a single-point residue mutation between ASFV-Georgia2010 and ASFV-Henin/2022 at position D51N, while there were no observable amino acid differences between ASFV-Georgia2010 and ASFV-Ghana2021 in protein C122R. Similarly, ASFV-Georgia2010 protein B119L presents a single point mutation, F18S, when compared with ASFV-Henin/2022, while there are no observed mutations in ASFV-Ghana2021.

Interestingly, the observed genetic changes in these 73 ASFV proteins are either identical in both ASFV-Ghana2021 and ASFV-Henin/2022, or the changes are more significant in ASFV-Ghana2021 than in ASFV-Henin/2022. In most cases, ASFV-Ghana2021 has all the same mutations in a particular protein as are found in ASFV-Henin/2022, plus additional mutations. This is interesting in terms of cross-protection, since the opposite result would be easily explainable. It is expected that the two overall most closely related viruses would have cross-protection (ASFV-G-ΔI177L and ASFV-Henin/2022), while the virus harboring many more mutations (ASFV-Ghana2021) would lack cross-protection; however, experimental results demonstrate that actually the opposite occurs.

Nevertheless, experimental evidence would have to be obtained to conclude that these small changes may have an effect on cross-protection, justifying the reported lack of cross-protection between the ASFV-Georgia2010 and the genotype 1-2 hybrid viruses.

However, there may be some additional causes that would explain the presence or absence of cross-protection in addition to just the linear comparison of mutations. The process inducing an immune response that allows for protection even between genetically close ASFV strains is not well characterized and likely could not be predicted solely on specific amino acid changes or genetic distance related to an individual protein or proteins. Hypothetically, it could be that rather than individual protein changes, particular combinations of changes in multiple proteins may alter the specificity of the induced immune response. It could be hypothesized that some protein changes could potentially alter individual protein structure or function perhaps inducing compensatory mutations in other ASFV proteins occurring during virus evolution. Thus, it could be possible that some of the additional changes observed in proteins of the Ghana2021 isolate over those in Henin/2022 isolate could revert either the structure or function of those proteins creating more antigenic/immunogenic similarities with the ASFV-Georgia2010 than the ASFV-Henin/2002 strain, thus explaining the cross-protection results observed with Ghana2021 and not with ASFV-Henin/2002.

Comparisons were also made with the other more diverse biotype strains but no conclusive evidence of the proteins involved in lack of cross-protection could be determined. That may be mostly due to the majority of proteins having genetic changes in all of these strains when compared to ASFV-Georgia2010, with only one protein showing solid conservation in all isolates tested in this study, A104R.

This study further shows that it is necessary to classify ASFV based not on the analysis of nucleotide sequences in limited genomic areas, as genotyping was performed in the past [34], but by taking into account all ASFV ORFs and biotyping the different ASFV strains. Interestingly, biotyping predicts that viruses in Biotype 1 and Biotype 2 are closely related when they are compared with viruses belonging to other biotypes. In addition, ASFV hybrid strains of Biotype 1-2 are a different subset of strains in regard to cross-protection than Biotype 1 or Biotype 2. However, more experimental data between other closely related biotypes will be necessary to help understand the limits of predicting cross-protection. Ultimately, to date the classification of ASFV by biotypes [28] has the highest potential to predict the probable presence/absence of cross-protection of old or newly emerging strains of ASFV by genomic analysis. So far, the very limited available information reported here indicates the presence of cross-protection among viruses within Biotype 1 or Biotype 2 [18,35]. Previous studies support the presence of cross-protection between Biotype 1 and 2 viruses, as similarly shown in this report [22], suggesting that closely related biotypes could have overlapping cross-protection profiles thus potentially favoring the use of single vaccine candidates.

Results reported here indicate that while using a single or small subset of ASFV proteins for the classification of ASFV whether by looking at nucleotide sequences [34], as has been traditionally conducted in the past, or more recently, using amino acid sequences [27,36], are useful for outbreak tracking, these methods do not have any meaning for predicting the potential of cross-protection, as both methods show all Biotype 1-2 hybrids as belonging to genotype I. However, this hybrid strain, which evolved between an improperly used and tested vaccine from the 1950’s and the recent circulating strain of ASFV Biotype 2, when characterized by a global protein analysis, remains a separate biotype than Biotype 1 or Biotype 2.

The results presented here and in other studies may support the concept that cross-protection between ASFV isolates is not based on the individual protein scale, and could rely, at least partially, on protein complexes that may occur during infection. This hypothesis would further explain the results presented here with cross-protection of ASFV-G-ΔI177L with ASFV Ghana2021 and those already published with the ASFV-Henin2021 strain. This hypothesis could also explain the lack of positive results in terms of the development of effective subunit vaccines for ASFV, mostly based on the use of individual proteins as immunogens [37,38,39,40]. All those attempts have relied on the use of groups of individual proteins in various expression vectors, and not necessarily on ASFV protein complexes that are formed during ASFV infection with live-attenuated ASFV vaccines.

To date the only effective ASFV vaccines developed are those based on the use of live-attenuated ASFV strains and, until the protective antigens are discovered and properly delivered, live-attenuated vaccines matched by biotype will likely be the only available rational approach for deciding vaccination procedures to control and manage ASFV outbreaks.

## 5. Conclusions

This report constitutes one of the few studies presenting data on cross-protection by a live-attenuated vaccine strain against a variety of genetically distant virulent field isolates, belonging to different biotypes, using a standardized challenge model. Results presented here support that cross-protection results cannot be predicted solely based on the classical methodology for genotyping ASFV field isolates. Although biotyping appears to be more accurate, it is clear that, at this point in ASFV vaccine research, the presence of cross-protection can only be demonstrated by the experimental challenge of vaccinated animals, particularly between biotypes. However, additional gathering of experimental data, especially considering the cross-protection observed between Biotype 2 and 1, and the lack of cross-protection between Biotype 2 and the hybrid Biotype 1/2 strains observed by others, may lead to more refined prediction of cross-protection using more sophisticated genomic tools that take into account all open reading frames of ASFV, such as biotyping, and the intricate pattern of ORF expression.

## 6. Patents

Manuel Borca and Douglas Gladue have a patent on the ASFV-G-ΔI177L Vaccine USA patent number: 11007263B2.

## Figures and Tables

**Table 1:** Summary of temperature and viremia after vaccination for all the groups of vaccinated animals with ASFV-G-ΔI177L.

	Fever Post-Vaccination			Viremia (HAD_50_) Post-Vaccination		
Group (Biotype)	Days to Onset	Duration (Days)	Animals with Fever	Days to Onset	Duration (Days)	Animals Viremic
Georgia10 (2)	13.50 (±5.50)	1.00 (±0.00)	2/6	13.50 (±5.50)	1.00 (±0.00)	2/6
Ghana2021 (1)	10.50 (±0.50)	1.25 (±0.25)	4/6	10.50 (±0.50)	1.25 (±0.25)	4/6
Malawi Lil20 (6)	11.25 (±2.29)	1.00 (±0.00)	4/4	11.25 (±2.29)	1.00 (±0.00)	4/4
Pret 4 (3)	7.33 (±0.67)	2.67 (±1.67)	3/6	7.33 (±0.67)	2.67 (±1.67)	3/6
Kenya1033 (5)	12.00 (±1.76)	2.20 (±0.80)	5/6	12.00 (±1.76)	2.20 (±0.80)	5/6
Uganda65 (4)	11.67 (±1.05)	1.67 (±0.42)	6/6	11.67 (±1.05)	1.67 (±0.42)	6/6

**Table 2:** Summary of temperature and viremia after challenge for all the groups of animals vaccinated with ASFV-G-ΔI177L or mock-vaccinated.

				Fever (>40 °C) Post-Challenge			Viremia (HAD_50_) Post-Challenge		
Vacc.	Challenge	Num. Survivors	Euthanasia (Day)	Num. Days to Onset	Duration (Days)	Num. Fever	Num. Days to Onset	Duration (Days)	Num. Viremic
YES	Georgia10	5/5 (100%)	21.0 (+/−0.0)	13.0 (±0.0)	5.0 (±0.0)	1/5	15.33 (+/−5.67)	1.20 (+/−0.73)	3/5
NO	Georgia10	0/5 (0%)	6.4 (+/−0.4)	4.4 (±0.2)	3.0 (±0.5)	5/5	4.00 (+/−0.00)	3.40 (+/−0.40)	5/5
YES	Ghana2021	4/5 (80%)	20.6 (+/−0.4)	6.8 (±0.8)	2.8 (±0.5)	4/5	11.00 (+/−0.00)	1.80 (+/−1.80)	1/5
NO	Ghana2021	0/5 (0%)	7.4 (+/−0.2)	4.8 (±0.2)	3.6 (±0.2)	5/5	4.00 (+/−0.00)	4.40 (+/−0.24)	5/5
YES	MalawiLil20	0/5 (0%)	8.4 (+/−1.2)	5.6 (±0.6)	2.8 (±0.4)	5/5	6.00 (+/−1.00)	4.40 (+/−1,63)	3/5
NO	MalawiLil20	0/5 (0%)	7.0 (+/−0.0)	4.2 (±0.2)	2.8 (±0.2)	5/5	4.00 (+/−0.00)	3.00 (+/−0.00)	5/5
YES	Pret4	0/5 (0%)	10.8 (+/−2.4)	6.6 (±1.9)	5.2 (±1.2)	5/5	7.50 (+/−2.02)	3.00 (+/−1.10)	4/5
NO	Pret4	0/5 (0%)	7.8 (+/−1.1)	5.6 (±0.9)	3.0 (±1.0)	5/5	4.00 (+/−0.00)	4.80 (+/−1.11)	5/5
YES	Ken1033	0/5 (0%)	5.8 (+/−0.4)	3.8 (±0.4)	3.0 (±0.0)	5/5	4.00 (+/−0.00)	2.80 (+/−0.37)	5/5
NO	Ken1033	0/5 (0%)	5.6 (+/−0.6)	3.0 (±0.0)	3.6 (±0.6)	5/5	4.00 (+/−0.00)	2.60 (+/−0.60)	5/5
YES	Uganda65	0/5 (0%)	5.6 (+/−0.2)	4.0 (±0.3)	2.6 (±0.2)	5/5	4.00 (+/−0.00)	2.60 (+/−0.24)	5/5
NO	Uganda65	0/5 (0%)	5.0 (+/−0.0)	3 (±0.0)	3.0 (±0.0)	5/5	4.00 (+/−0.00)	2.00 (+/−0.00)	5/5

“Vacc.” stands for vaccinated, and “Num.” stands for number.

## Data Availability

All data is included in this manuscript.

## Supplementary Materials

The following supporting information can be downloaded at https://www.mdpi.com/article/10.3390/vaccines13080858/s1: Table S1: Protein changes by Isoalte.
